# Biochemical Basis of *E. coli* Topoisomerase I Relaxation Activity Reduction by Nonenzymatic Lysine Acetylation

**DOI:** 10.3390/ijms19051439

**Published:** 2018-05-11

**Authors:** Qingxuan Zhou, Mario E. Gomez Hernandez, Francisco Fernandez-Lima, Yuk-Ching Tse-Dinh

**Affiliations:** 1 Biomolecular Sciences Institute, Florida International University, Miami, FL 33199, USA; qzhou004@fiu.edu (Q.Z.); fernandf@fiu.edu (F.F.-L.); 2 Department of Chemistry and Biochemistry, Florida International University, Miami, FL 33199, USA; gomezmar@fiu.edu

**Keywords:** topoisomerase, lysine acetylation, DNA supercoiling, DNA–protein interaction, acetyl phosphate, posttranslational modification

## Abstract

The relaxation activity of *E. coli* topoisomerase I is required for regulation of global and local DNA supercoiling. The in vivo topoisomerase I enzyme activity is sensitive to lysine acetylation–deacetylation and can affect DNA supercoiling and growth as a result. Nonenzymatic lysine acetylation by acetyl phosphate has been shown to reduce the relaxation activity of *E. coli* topoisomerase I. In this work, the biochemical consequence of topoisomerase I modification by acetyl phosphate with enzymatic assays was studied. Results showed that noncovalent binding to DNA and DNA cleavage by the enzyme were reduced as a result of the acetylation, with greater effect on DNA cleavage. Four lysine acetylation sites were identified using bottom-up proteomics: Lys13, Lys45, Lys346, and Lys488. The Lys13 residue modified by acetyl phosphate has not been reported previously as a lysine acetylation site for *E. coli* topoisomerase I. We discuss the potential biochemical consequence of lysine acetylation at this strictly conserved lysine and other lysine residues on the enzyme based on available genetic and structural information.

## 1. Introduction

The supercoiling level of DNA is important for vital cellular processes including transcription, replication, and recombination [1,2,3]. The maintenance of DNA supercoiling levels in bacteria relies largely on the negative supercoiling action of DNA gyrase and the relaxation action of DNA topoisomerase I [4,5]. Hypernegative DNA supercoiling has been demonstrated to result in RNA/DNA hybrids (R-loop) formation that can affect both transcription elongation and replication, and consequently lead to genomic instability in bacteria [6,7,8]. Type IA DNA topoisomerase I is a ubiquitous enzyme present in every bacterium [9,10] and is responsible for removing transcription-driven negative DNA supercoiling and suppression of R-loops during transcription elongation [11,12,13,14]. In addition, topoisomerase I function is required for rapid transcription of stress response genes and cellular viability following stress challenges including heat shock, acid, and oxidative stresses [15,16,17,18,19].

*Escherichia coli* topoisomerase I, encoded by *topA*, relaxes negative DNA supercoiling by transiently cleaving the G-strand of underwound duplex DNA, transferring the T-strand and rejoining the G-strand [20]. In earlier work, we have demonstrated that the catalytic activity of *E. coli* topoisomerase I is modulated by the posttranslational modification of lysine acetylation–deacetylation, and the protective effect from excessive acetylation by deacetylase CobB has a global impact on DNA supercoiling and cell growth [21]. Previous proteomics studies have shown multiple lysine residues of *E. coli* topoisomerase I to be acetylated under various growth conditions [22,23,24,25] when cell lysates were analyzed. Most acetylated lysine residues are located on solvent exposed positions, suggesting the potential impact of lysine acetylation on topoisomerase I interactions with chromosomal DNA and with other proteins, or potentially on interactions between topoisomerase I subdomains. Hence, the elucidation of the mechanism of topoisomerase I regulation by lysine acetylation–deacetylation is of significance.

Deacetylase CobB, which interacts directly with topoisomerase I [21,26], can reverse both enzymatic and nonenzymatic lysine acetylation [27]. Acetyl phosphate has been suggested to be a critical determinant of lysine acetylation in *E. coli* [24]. Here, we demonstrated that in vitro nonenzymatic lysine acetylation of topoisomerase I by acetyl phosphate affects the binding and cleavage of single-stranded DNA by *E. coli* topoisomerase I. We chose the concentration of 5 mM acetyl phosphate because a previous study has suggested that the intracellular concentration of acetyl phosphate can be as high as 4.5 mM for *E. coli* [28]. Bottom-up proteomics using tryptic digest of topoisomerase I modified by acetyl phosphate treatment allowed the observation of the acetylated lysine residues. The acetylation positions in the tryptic peptides were confirmed using tandem mass spectrometry. We identified Lys13 as a new acetylated lysine site. This residue has been shown in a previous site-directed mutagenesis study to be required for single-stranded DNA cleavage by *E. coli* topoisomerase I [29]. Acetylation of the positively charged side chain at this and other lysine residues in topoisomerase I is expected to collectively suppress the binding and cleavage of single-stranded DNA by topoisomerase I and reduce the enzyme relaxation activity.

## 2. Results

### 2.1. Reduction of Topoisomerase I Relaxation Activity Following Nonenzymatic Acetylation

**Figure 1:**
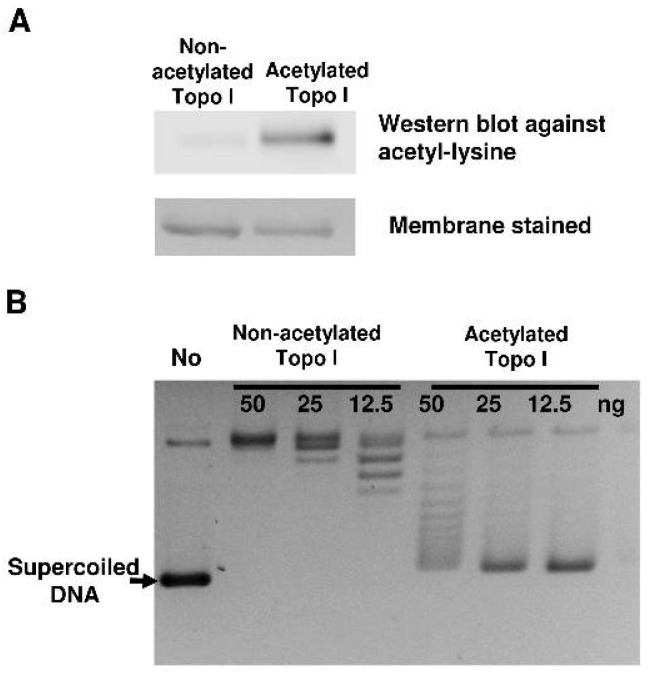
Characterization of topoisomerase I acetylated by acetyl phosphate. (**A**) Western blot analysis of acetylated topoisomerase I. Purified *E. coli* topoisomerase I (1 μg) was incubated with 5 mM acetyl phosphate at 37 °C for 4 h. Acetylation of topoisomerase I was visualized by Western blotting using an anti-acetyl lysine antibody. Topoisomerase I on the membrane was stained with Coomassie blue. (**B**) Serial dilutions of nonacetylated and acetylated topoisomerase I were incubated with 180 ng of negatively supercoiled plasmid DNA at 37 °C for 30 min to assay relaxation activity. No: no enzyme.

In a previous study [21], we showed that in vitro acetyl-phosphate-mediated nonenzymatic lysine acetylation reduced the catalytic activity of topoisomerase I. Incubation of 5 mM acetyl phosphate with recombinant topoisomerase I increased the topoisomerase I lysine acetylation level observed by Western blotting with antibodies against acetyl lysine (Figure 1A). A greater than fourfold reduction in topoisomerase I catalytic activity can be observed by relaxation assay with the negatively supercoiled DNA substrate (Figure 1B). We have previously reported an approximately eightfold reduction in topoisomerase I relaxation activity following treatment with 5 mM acetyl phosphate [21]. We hypothesized that acetylation on lysine residues reduced topoisomerase I catalytic activity via affecting specific steps in the catalytic cycle.

### 2.2. Decreased DNA Cleavage by Acetylated Topoisomerase I

**Figure 2:**
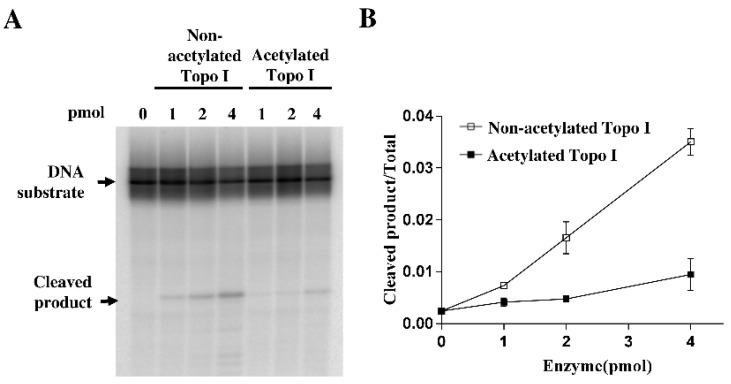
Cleavage assay of nonacetylated and acetylated *E. coli* topoisomerase I. (**A**) The indicated amount of nonacetylated topoisomerase I and acetylated topoisomerase I were incubated with 5’-end labeled 59-base oligonucleotide substrate at 37 °C for 30 min. The reactions were stopped and electrophoresed in 15% sequencing gel. The level of cleaved product and DNA substrate were analyzed by PhosphorImager. (**B**) Quantification of the fraction of cleaved product from three experiments is shown. Error bar indicates standard deviation (*n* = 3).

The catalytic activity of *E. coli* topoisomerase I requires a number of partial steps: the noncovalent binding of topoisomerase I to the single-stranded region of the DNA substrate, the cleavage of single-stranded DNA, and religation of the cleaved DNA strand after strand passage. To determine whether the cleavage step during the catalytic cycle is affected by acetylation, a single-stranded oligonucleotide DNA labeled with ^32^P at 5′-end was used as the substrate for incubation with 1 pmol, 2 pmol, and 4 pmol of nonacetylated and acetylated topoisomerase I. Acetylated topoisomerase I showed approximately fourfold reduced cleavage activity compared to nonacetylated topoisomerase I (Figure 2). Thus, the reduced cleavage activity of acetylated topoisomerase I could partially account for the reduction in enzyme relaxation activity following acetylation by acetyl phosphate.

### 2.3. Acetylation of Topoisomerase I Reduced Binding Affinity of the DNA Substrate

**Figure 3:**
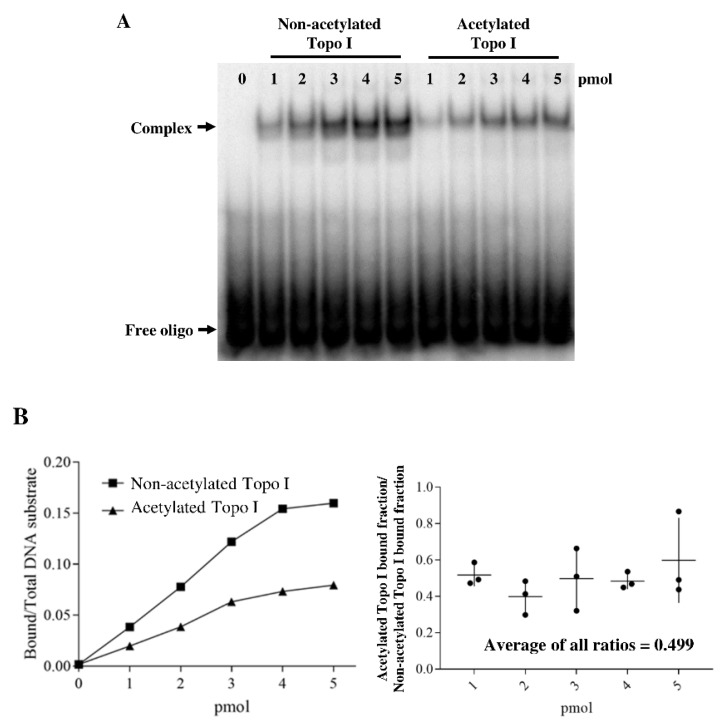
Gel shift assay of nonacetylated and acetylated *E. coli* topoisomerase I. (**A**) The indicated amounts of nonacetylated and acetylated topoisomerase I were incubated with labeled 59-base oligonucleotide at 37 °C for 5 min. The reactions were electrophoresed in 6% native polyacrylamide gel. The relative level of enzyme–oligo complex and free oligo was analyzed by PhosphorImager. (**B**) Quantitation of the fraction of total DNA substrate bound by topoisomerase I. The ratio of the bound fraction for acetylated topoisomerase I versus bound fraction for nonacetylated topoisomerase I at each protein concentration was determined for three replicate experiments and shown as dots in the right panel.

We next investigated whether the topoisomerase I binding affinity for the DNA substrate is affected by acetylation using the gel shift assay. ^32^P-labeled oligonucleotide substrate was incubated with increasing amounts of either nonacetylated or acetylated topoisomerase I. Our result showed that acetylation by acetyl phosphate reduced the noncovalent binding of DNA to topoisomerase I (Figure 3). The effect of acetylation on DNA binding by topoisomerase I at the different protein:DNA ratios averaged to be ~2-fold decrease from 3 replicates of the gel shift assay (Figure 3B). Therefore, the reduction in topoisomerase I relaxation and DNA cleavage activity cannot be attributed singly to decreased level of noncovalent enzyme–DNA complex formed at the start of the catalytic cycle.

### 2.4. Identification of Lysines in Topoisomerase I Modified by Nonenzymatic Acetylation

**Figure 4:**
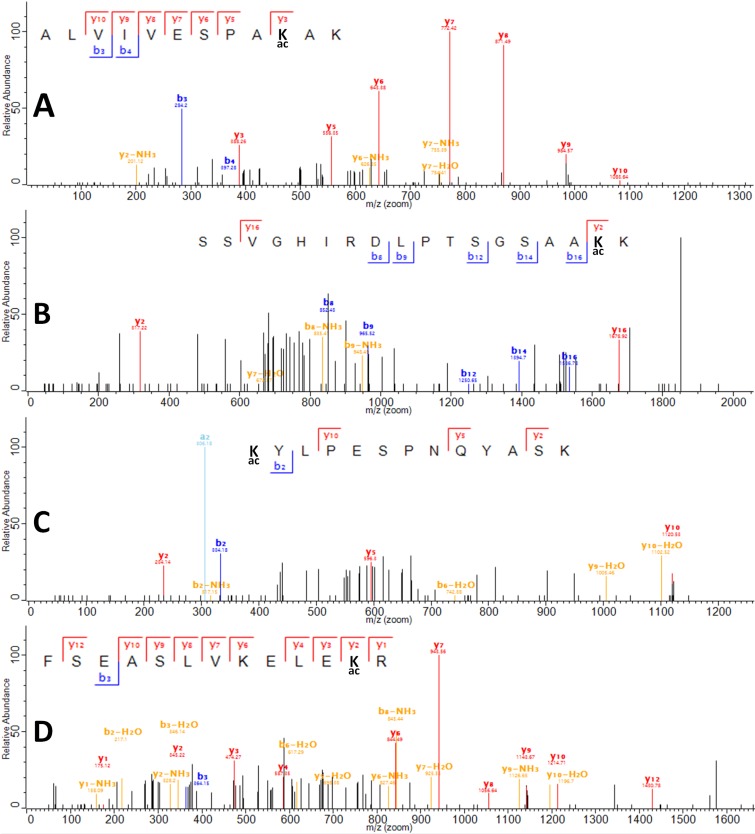
Collision induced dissociation (CID) spectra of lysine acetylation sites 13, 45, 346, 488. Peaks with masses and assignments are shown for (**A**) Sequence for Position Lys13: ALVIVESPAK(Ac)AK; (**B**) Sequence for position Lys45: SSVGHIRDLPTSGSAAK(Ac)K; (**C**) Sequence for position Lys346: K(Ac)YLPESPNQYASK; (**D**) Sequence for position Lys488: FSEASLVKELEK(Ac)R. Red labels denote y-ion series, blue labels denote b-ion series. Other ions are denoted with yellow and light blue labels.

**Figure 5:**
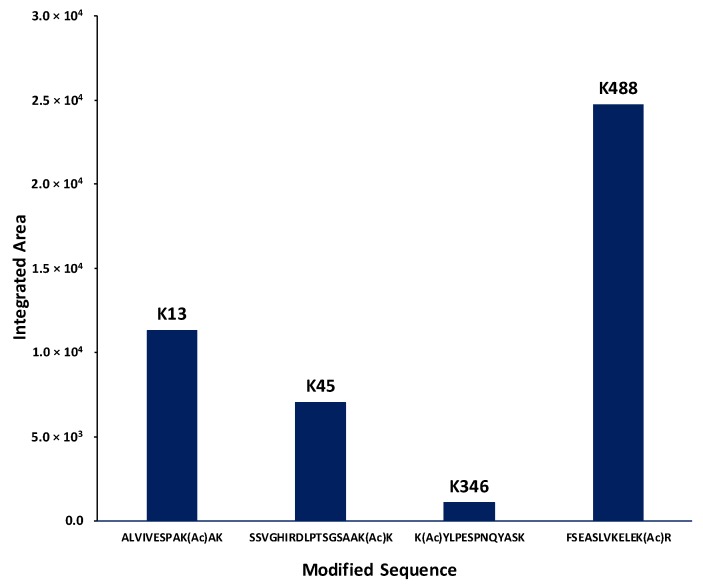
Comparison of the extracted ion chromatogram (EIC) integrated areas for the detected acetylated peptides. The comparison of the integrated EIC areas for the four peptides with identified lysine acetylation sites suggests that the abundance of positions Lys488 and Lys13 are higher than Lys45 and Lys346.

Collision-induced dissociation quadrupole time-of-flight tandem mass spectrometry (CID, Q-TOF-MS) analysis of the tryptic digest of acetylated topoisomerase I detected 60 unique tryptic peptides (Table S1) for a sequence coverage of 58.6%, of which 4 peptides were determined to contain acetylated lysines using tandem MS analysis (ALVIVESPAK(Ac)AK, SSVGHIRDLPTSGSAAK(Ac)K, K(Ac)YLPESPNQYASK, and FSEASLVKELEK(Ac)R) at positions Lys13, Lys45, Lys346, and Lys488 (Figure 4). The detected acetylated peptides were assigned MaxQuant scores >30 (MaxQuant evidence can be found in Table S2). In addition, the integrated areas of the extracted ion chromatograms (EIC) suggest that the abundance for the peptides containing positions Lys45 and Lys346 are lower than the abundance of the peptides containing positions Lys13 and Lys488 (Figure 5). Thus, the scores of assignment for positions 45 and 346 are reported at 31 and 33, respectively, whereas for positions Lys13 and Lys488, the scores of assignment are 75 and 68, respectively. In terms of the probability of acetylation site assignment, positions Lys13, Lys45, and Lys346 have probabilities values of 1, whereas position Lys488 in the peptide FSEASLVKELEKR has a probability value of 0.9, while the other potential acetylation site at Lys484 has a probability value of 0.09 within the same observed peptide. Thus, MaxQuant analysis locates the acetylation at position Lys488. For experimental control purposes, bottom-up proteomic analysis of the nonacetylated topoisomerase I detected 34 unique tryptic peptides for a sequence coverage of 43.7%, without detecting any acetylated peptide.

## 3. Discussion

In this study, we investigated the biochemical basis for the reduction of *E. coli* topoisomerase I relaxation activity following nonenzymatic lysine acetylation by acetyl phosphate. It has been shown that in *E. coli*, nonenzymatic acetyl-phosphate-dependent lysine acetylation is a major mechanism for this posttranslational modification of cellular proteins [24,30]. The positive charge on the lysine side chains are expected to contribute to DNA binding by electrostatic interaction with the negatively charged DNA phosphates. We found that noncovalent binding of DNA by topoisomerase I was decreased about twofold by lysine acetylation. The reduction of noncovalent DNA binding from acetylation would lead to reduced relaxation activity because of noncovalent binding of the DNA substrate is the first step for the relaxation of supercoiled DNA by topoisomerase I. However, the effect of acetyl phosphate treatment on DNA cleavage and DNA relaxation is greater than the effect observed on noncovalent DNA binding. This suggests that there may be additional effects from lysine acetylation on the catalytic steps following initial DNA binding. We did not assay the DNA religation activity of the acetylated topoisomerase I because of the low level of DNA cleavage product obtained.

**Figure 6:**
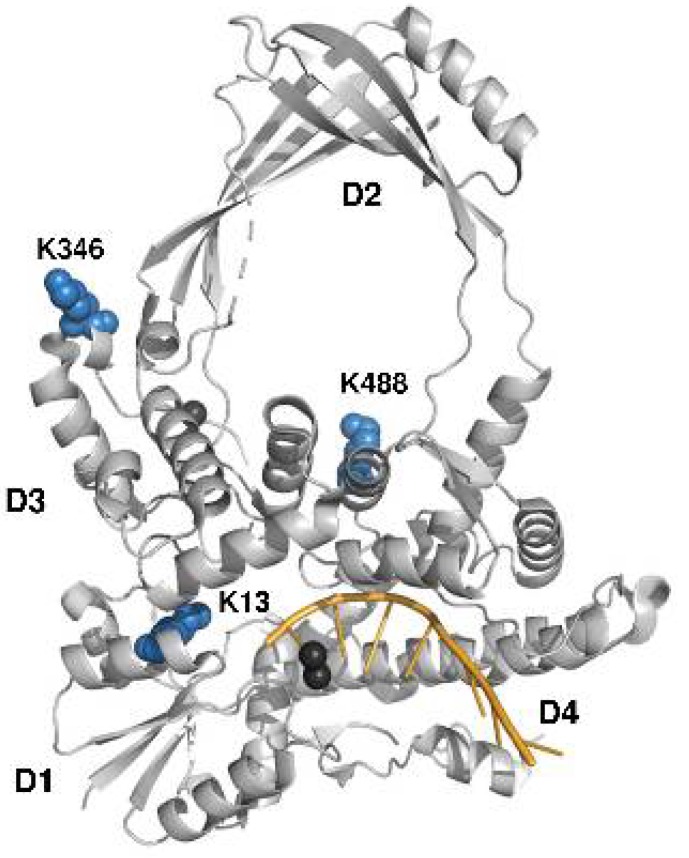
Positions of lysines identified to be acetylated by acetyl phosphate. The identified lysine residues (highlighted in blue) are shown in the structure of the covalent complex between *E. coli* topoisomerase I N-terminal domain and cleaved oligonucleotide substrate (highlighted in orange) (PDB: 3PX7). Lys45 is part of a disordered region near the bottom of subdomain D4 that is not observable in the crystal structure. The figure is made with Pymol.

We identified four positions of acetyl phosphate mediated lysine acetylation by bottom-up proteomics. Furthermore, data analysis of the mass spectrometry results indicates that for the acetylated topoisomerase I peptides, the abundances of the peptides containing acetylated lysines at positions Lys13 and Lys488 are higher than those containing acetylation at positions Lys45 and Lys346. Lys45 is located in a disordered region near the bottom of subdomain D4 that cannot be observed in the available topoisomerase I crystal structures [31,32,33]. Lys346 is a surface-exposed lysine in subdomain D3 (Figure 6). Lys488 is located at the interface between subdomains D3 and D4 near the entrance to the interior hole of the topoisomerase I structure. It may be involved in the interaction with the passing strand of DNA during the relaxation of supercoiled DNA by topoisomerase I or the conformational change required to allow access of the passing strand into the interior hole.

In this work, we report the acetylation of *E. coli* topoisomerase at position Lys13 for the first time. Other reports on whole proteome analysis of *E. coli* lysine acetylation have not observed the acetylation of Lys13 probably due to gaps in the sequence coverage obtained during mass spectrometry analysis using trypsin to conduct the enzymatic digest of the whole proteome. A previous study has shown by site-directed mutagenesis that mutation of Lys13 to either alanine or arginine [29] greatly reduced the DNA relaxation and cleavage activity of *E. coli* topoisomerase I. The alanine substitution removes the positive charge from the Lys13 side chain similar to loss of positive charge from acetylation. The arginine substitution maintains the positive charge of the side chain, but the bulkier size of the arginine side chain may account for the previously observed unfavorable noncovalent binding to DNA [29]. Acetylation of *E. coli* topoisomerase I Lys13 in vivo could result in immediate decrease of its relaxation activity unless the acetylation modification is reversed by the action of the deacetylase CobB. The potential regulation of topoisomerase I activity and DNA supercoiling by the acetylation stoichiometry of Lys13 under different growth conditions should be further examined in future studies by quantitative mass spectrometry analysis designed to provide the maximum coverage of topoisomerase I.

There may be additional lysine residues on topoisomerase I that become acetylated following treatment with acetyl phosphate that were not found in the tryptic digest proteomics. We expect the inhibitory effects from acetylation of each lysine residue to be accumulative. Therefore, even though an individual lysine may only be acetylated in a relatively small percentage of the topoisomerase I polypeptides, a significant portion of the topoisomerase I polypeptides may have multiple lysine acetylation modifications, resulting in reduction of the overall topoisomerase I relaxation activity. We would try to increase coverage of mass spectrometry analysis in further studies of topoisomerase I modification by lysine acetylation in order to identify additional lysine acetylation sites that may be relevant for the catalytic activity. Site-directed mutagenesis involving changes in two and more lysine residues at a time would provide more insights into the biochemical consequence of acetyl phosphate modification of topoisomerase I.

It has been shown that while lysine acetylation by acetyl phosphate occurs at a low level in *E. coli*, the nonenzymatic lysine acetylation can still be a critical determinant of for bacterial protein acetylation [24] that might change dynamically in response to changes in metabolism and growth conditions. Proteins that interact with nucleic acids are often rich in lysine residues. Previous studies have shown that nonenzymatic acetylation occurs at other DNA binding proteins of bacteria. For instance, acetyl phosphate driven acetylation at Lys154 of the *E. coli* transcription regulator RcsB inhibits its function [34], and the acetylation can be reversed by CobB deacetylase. Other DNA binding proteins found as acetylated proteins in *E. coli cobB* mutant strain include RNA polymerase, IHF, SeqA, Hu, Fis, and DNA gyrase [27]. Although regulation of DNA supercoiling by CobB can potentially be mediated by deacetylation of Hu, Fis, and DNA gyrase, these proteins have not been shown experimentally to interact directly with CobB. In contrast, direct protein–protein interaction between *E. coli* CobB and topoisomerase I has been demonstrated experimentally using proteome microarray, pulldown, and Streptavidin biosensors [21,26]. The potential selective stimulation of topoisomerase I by CobB in vivo is in agreement with the increase in DNA supercoiling observed in the *cobB* mutant, as well as the partial rescue of the slow growth phenotype of the *cobB* mutant by overexpression of recombinant topoisomerase I [21].

## 4. Materials and Methods

### 4.1. Acetyl Phosphate Treatment of E. coli Topoisomerase I

Purified *E. coli* topoisomerase I [35] was incubated at 0.1 μg/μL with 5 mM acetyl phosphate at 37 °C in previously published conditions [21,36] of 150 mM Tris-HCl (pH 8.0), 10% glycerol, and 10 mM MgCl_2_ for 4 h. The enzyme was either assayed immediately for topoisomerase enzyme activity or mixed with an equal volume of 2XSDS loading buffer for sodium dodecyl sulfate polyacrylamide gel electrophoresis (SDS-PAGE). Western blot analysis of lysine acetylation was carried out using mouse monoclonal anti-acetyl lysine antibody (Cell Signaling Technology (Danvers, MA, USA)).

### 4.2. Topoisomerase I Relaxation Activity Assay

To assay the relaxation activity of topoisomerase I, each protein was serially diluted as indicated and incubated with 180 ng of supercoiled pBAD/Thio plasmid DNA in 20 μL reaction (10 mM Tris-HCl, pH 8.0, 50 mM NaCl, 0.1 mg/mL gelatin, 6 mM MgCl_2_). After incubation at 37 °C for 30 min, the reaction was stopped by addition of 4 μL stop solution (50 mM Ethylenediaminetetraacetic acid, 50% glycerol, 0.5% (*v*/*v*) bromophenol blue). The plasmid DNA was electrophoresed in 1% agarose gel with TAE buffer (40 mM Tris-acetate, pH 8.3, 2 mM EDTA) and visualized with ultraviolet light.

### 4.3. DNA Cleavage Assay

A 59-base oligonucleotide DNA substrate [37] (5′-GCCCTGAAAGATTATGCAATGCGCT↓TTGGGCAAACCAAGAGAGCATAATCTTTCAGGGC-3′, (↓ representing the cleavage site) was labeled with ^32^P at the 5′-end with T4 polynucleotide kinase and used in the cleavage assays. For the cleavage assay, ^32^P-labeled oligonucleotide substrate was added to nonacetylated or acetylated topoisomerase I in 5 µL acetylation reaction buffer along with 10 mM EDTA to favor DNA cleavage over religation. Following incubation at 37 °C for 30 min, the reactions were stopped by addition of 5 µL of stop solution (79% formamide, 0.2M NaOH, 0.04% bromophenol blue). The samples were heated at 95 °C for 5 min and electrophoresed in 15% sequencing gel with 1 X TBE buffer (90 mM Tris-borate, pH 8.0, 2 mM EDTA), followed by analysis with the Pharos FX Plus Phosphorimager (Bio-Rad (Hercules, CA, USA)). The fraction of cleaved oligonucleotide product was quantified by Quantity One 1-D analysis.

### 4.4. DNA Binding Assay

Gel shift assay was used to measure DNA binding. The ^32^P-labeled 59-base oligonucleotide DNA substrate was incubated with nonacetylated and acetylated *E. coli* topoisomerase I in 10 µL reaction (150 mM Tris-HCl, pH 8.0, 1 mg/mL BSA, 10% glycerol, 5 mM MgCl_2_, 10 mM EDTA). After incubation at 37 °C for 5 min, the DNA–enzyme complex was separated from the unbound DNA by electrophoresis in 6% native polyacrylamide gel with 0.5× TBE buffer (45 mM Tris-borate, pH 8.0, 1 mM EDTA). The fraction of oligonucleotide bound to the enzyme was determined by analysis with the Pharos FX Plus Phosphorimager.

### 4.5. In-Gel Tryptic Digestion

Fifteen micrograms of acetylated topoisomerase I and 15 μg of nonacetylated topoisomerase I were electrophoresed in 10% SDS-PAGE gel and visualized by Coomassie blue staining. The sample bands were excised, divided into two parts, and placed in a clean plastic Eppendorf tube prior to in-gel digestion with trypsin. In-gel trypsin digestion was conducted utilizing the Thermo Scientific (Rockford, IL, USA) digestion kit and protocol number 89871. In summary, each gel band was first destained in ammonium bicarbonate in acetonitrile/water. Subsequently, the gel pieces were reduced utilizing a Tris[2-carboxyethyl]phosphine (TCEP) reducing buffer and alkylated with iodoacetamide in water. Thereafter, the gel pieces were washed with an ammonium bicarbonate destaining solution twice. Digestion was then conducted by first shrinking the gel pieces with 50 µL of acetonitrile with an incubation time of 15 min at room temperature. The acetonitrile was then removed and the gel pieces were allowed to airdry for 10 min. The gel pieces were then covered with 10 µL of the trypsin solution and incubated at room temperature for 15 min. Finally, 25 µL of the digestion buffer (2 mg/mL ammonium bicarbonate, approx. 25.3 mM) were added to the gel pieces and the samples were incubated for 14 h at 30 °C in a water bath. The supernatant was then separated from the gel pieces and placed in a clean Eppendorf tube. Immediately after, an additional extraction of peptides was performed by adding 10 µL of 1% formic acid to the gel band and allowed to incubate for an additional 10 min. The supernatant was combined with the gel peptide extract and used for mass spectrometry analysis.

### 4.6. Bottom-Up Proteomics Analysis

Proteomics analysis of the trypsin digested nonacetylated and acetylated topoisomerase I enzymes was conducted using liquid chromatography coupled to tandem mass spectrometry. A Bruker Impact HD ESI-QTOF-MS (Billerica, MA, USA) instrument operated coupled to a Prominence LC-20CE Ultra-Fast Liquid Chromatograph (Shimadzu, Kyoto, Japan) was used for all the analyses. A Waters XBridge Peptide BEH C18 (300 Å, 5 μm, 4.6 × 250 mm) column protected by a 4.6 × 20 mm guard column was used. Peptides were eluted by a 46 min binary gradient with a mobile phase consisting of 0.1% formic acid in water (mobile phase A) and 0.1% formic acid in acetonitrile (mobile phase B) run according to the following timetable: hold 10% B for 5 min diverting to waste; hold 10% B for 2.6 min infusing to the mass spectrometer; ramp to 25% B in 10.6 min; ramp to 30% B in 2.8 min; ramp to 35% B in 6.3 min; ramp to 100% B for 1.7 min; hold 100% B for 4 min then divert to waste; hold 100% B for 2 min; return to 10% B in 5 min; hold 10% B for 6 min for re-equilibration. Flow rate was constant at 0.4 mL/min and oven temperature kept constant at 50 °C. Tandem mass spectrometry data was analyzed utilizing the MaxQuant proteomics analysis suite applying a false discovery rate (FDR) of 1% and a minimum score for modified peptides of 30, and MS/MS searches were conducted applying a 0.5 Da tolerance. For the digest search parameters, trypsin/p was utilized as the digest enzyme allowing up to six missed cleavages. For variable modifications, oxidation of methionine (M) and acetylation of the protein N-term and lysine (K) residues were applied as search parameters, whereas carbamidomethylation of cysteine (C) was applied as the sole fixed modification. In addition, the integrated areas for the extracted ion chromatograms were calculated utilizing Bruker Daltonics Data Analysis version 5.1 with a tolerance of ±0.005 Da for the following masses: 634.3846, 783.8936, 789.4303, and 926.9974 for the ions containing Lys13, Lys346, Lys488, and Lys45, respectively. Finally, the area integration was conducted with an intensity threshold of 1000 and a sensitivity of 99% for all aforementioned masses.

## 5. Conclusions

Nonenzymatic lysine acetylation mediated by acetyl phosphate has been shown to reduce *E. coli* topoisomerase I relaxation activity. Here, we demonstrated the biochemical basis for this activity reduction. Noneyzmatic lysine acetylation reduced the DNA binding and DNA cleavage of topoisomerase I, with greater effect on the latter. We identified Lys13 as new lysine acetylation site in addition to Lys45, Lys346, and Lys488 which have been reported previously.

## Supplementary Materials

Supplementary materials can be found at http://www.mdpi.com/1422-0067/19/5/1439/s1.

## Abbreviations

Topo I	Topoisomerase I
TCEP	Tris[2-carboxyethyl]phosphine
MS	Mass Spectrometry
EIC	Extracted Ion Chromatograms
Q-TOF	Quadrupole Time of Flight
CID	Collision Induced Dissociation
