# Supplementary material for: Biochemical Basis of E. coli Topoisomerase I Relaxation Activity Reduction by Nonenzymatic Lysine Acetylation
Source: Int J Mol Sci. 2018 May 11;19(5):1439. doi: 10.3390/ijms19051439 (PMC5983628; doi:10.3390/ijms19051439)
Supplement: Supplementary file 1 [file ijms-19-01439-s001.zip › Supplmentary Information.pdf]

Supplementary Information

Biochemical Basis of *E. coli* Topoisomerase I Relaxation Activity Reduction by Non-Enzymatic Lysine Acetylation

Qingxuan Zhou, Mario Gomez, Francisco Fernandez-Lima, Yuk-Ching Tse-Dinh*

Biomolecular Sciences Institute, Florida International University, Miami FL 33199, USA
Department of Chemistry and Biochemistry, Florida International University, Mami FL 33199, USA

*Author to whom correspondence should be addressed. Email: yukching.tsedinh@fiu.edu

Table S1. List of Tryptic Peptides Identified by Mass Spectrometry. (A) Acetylated topoisomerase I peptides; (B) Non-acetylated topoisomerase I peptides.

Table S2. MaxQuant Raw Data Evidence Table. (A) Acetylated topoisomerase I peptides. Table S2A shows the unmodified peptides and the corresponding acetylated peptides paired by color sets. Also included are the protein groups reported by MaxQuant showing the identification score and % sequence coverage. Topoisomerase I is highlighted in green for clarity purposes. Table S2A also shows peptides containing oxidized methionines. (B) Non-acetylated topoisomerase I peptides. Table S2B shows the unmodified peptides and no acetylated peptides. Also included are the protein groups reported by MaxQuant showing the identification score and % sequence coverage. Topoisomerase I is highlighted in green for clarity purposes. Table S2B also shows peptides containing oxidized methionines.
